# Women’s perceptions of the pain assessment and non-pharmacological pain relief methods used during labor: A cross-sectional survey

**DOI:** 10.18332/ejm/146136

**Published:** 2022-04-13

**Authors:** Arja Rantala, Mervi Hakala, Tarja Pölkki

**Affiliations:** 1 Research Unit of Nursing Science and Health Management, Faculty of Medicine, University of Oulu, Oulu, Finland; 2 Department of Children and Women, Oulu University Hospital, Northern Ostrobothnia Hospital District, Oulu, Finland; 3 Oulu University of Applied Sciences, Oulu, Finland; 4 Medical Research Center Oulu, Oulu University Hospital, Oulu, Finland

**Keywords:** labor, midwife, non-pharmacological methods, pain, quantitative research

## Abstract

**INTRODUCTION:**

The use of non-pharmacological pain relief methods and pain assessment scales during labor has received limited research attention. This study aimed to describe women’s perceptions of the pain assessment and non-pharmacological pain relief methods used during labor.

**METHODS:**

A descriptive, cross-sectional survey was conducted. A convenience sample of women (n=204) from one Finnish maternity ward participated in the study. Women who had given birth were asked to respond to a validated questionnaire between November 2018 and February 2019. The statistical significance of observed differences was analyzed using the chi-squared test.

**RESULTS:**

Less than half (46%) of the women who gave birth at the hospital were asked to assess the intensity of their pain on a pain assessment scale. The most commonly used non-pharmacological pain relief methods were encouragement (92%), the presence of a midwife (82%), and proper breathing technique that was taught by a midwife (81%). Aqua blisters (3%), reflexology (e.g. zone magnets, 5%), and music (9%) were the least commonly used non-pharmacological methods during labor. The participants’ experiences of fear and pain were significantly associated with the implementation of pain assessment.

**CONCLUSIONS:**

Women’s pain was rarely evaluated by using a certain pain assessment scale. In addition, non-pharmacological pain relief methods were inadequately used during labor. More specifically, methods that required midwives’ own personal contributions were rarely offered to the women.

## INTRODUCTION

Women experience varying degrees of pain during labor, ranging from minimal pain to extreme, and distressing, levels of pain^1,2^. This demonstrates how perceived pain is influenced by the intricate and subjective interaction of multiple physiological and psychosocial factors related to a woman’s individual construction of labor stimuli^3^. For this reason, the World Health Organization (WHO) has provided recommendations for high-quality pain management during childbirth^4^.

The pain experienced during labor can be measured by different pain assessment scales such as the Visual Analogue Scale (VAS)^1,5^. Numeral Rating Scale (NRS), Verbal Rating Scale (VRS) or McGill pain questionnaire (three components: VAS, verbal response scale, and present pain intensity scale)^1,6,7^. However, pain assessments are not always conducted in a structured way, i.e. pain might be assessed verbally without any pain assessment or may be based on a midwife’s evaluation of a woman’s pain^8^. This is not relevant because midwives can sometimes underestimate labor pain^5^. It is challenging to assess a woman’s pain during labor, as a pain assessment should match individual preferences for mode and timing during labor to provide accurate results^1,9^.

In addition to having knowledge of different pain assessment approaches, healthcare personnel should be aware of different pain relief methods^4^ because the use of an analgesic increases the risk of episiotomies and the duration of labor^10^. Non-pharmacological pain relief approaches, for example, can help women to relax and feel in control during childbirth, which will enable them to work actively with their physiological responses^11^. However, women are not always sure about their willingness to use pain relief methods during labor^12^, or there may be a wide gap between the use of pain relief methods and women’s needs^13^. A cross-sectional study by Almushait and Ghani^14^ revealed that hospital professionals knew about most non-pharmacological pain relief methods that are relevant for handling pain during labor; however, the results showed that most of these methods are not used in practice. The highest barriers for not using non-pharmacological pain relief methods were patients’ lack of knowledge to use them or strong beliefs towards analgesic. In addition, most barriers were interrelated^14^.

Non-pharmacological methods such as music^11,15^, aromatherapy^16^, breathing patterns^16^, hypnosis^17^, massages^16^, the use of sterile water in intra- or subdermal injections to the back^18^, and cold treatments such ice massages^15^ can be used to relieve pain during labor. In addition, supporting women during labor by midwives, or chosen company, are effective for giving positive childbirth experience^19^. These methods can also reduce the use of pharmacological analgesia^20^. According to a qualitative systematic review by Thomson et al.^11^, relaxation and massage techniques served as distractors to alleviate women’s pain related to contractions. In another study, Czech et al.^21^ compared non-pharmacological and pharmacological methods used in labor, reporting that the satisfaction of childbirth depends not only on the level of experienced pain, but also on the care provided during labor^21^. The results of these two studies dictate that the role and benefits of non-pharmacological methods of pain relief during labor cannot be ignored^14^. According to the latest Cochrane^1^ overview of systematic reviews concerning pain management in labor, acupuncture, relaxation, massage, and immersion in water may improve pain management during labor relative to standard care and may ultimately result in greater satisfaction with pain relief. However, it remains unclear whether hypnosis, biofeedback, sterile water injection, aromatherapy, transcutaneous electrical nerve stimulation (TENS), and parenteral opioids, are more effective than a placebo or other interventions for pain management during labor^1^. In Finland, 93% of the women used at least one pain relief method during labor, with 42% using some non-pharmacological pain relief method during labor; however, there is no clear knowledge on the non-pharmacological methods most commonly used^22^.

In summary, earlier studies have focused on the effectiveness of different pain relief methods^1,11,15,16,18,20,21^ and the views of healthcare providers regarding the use of non-pharmacological pain relief methods during labor^14,16,23^. Based on our knowledge, there is a research gap concerning how women perceive the pain assessment and pain relief approaches they are subjected to while giving birth. Thus, the current study aims to describe women’s perceptions of the pain assessment and non-pharmacological pain relief methods that are used during labor.

Our research questions were as follows:

1. How was women’s pain assessed during labor?
2. Which non-pharmacological pain relief methods were used to alleviate women’s pain during labor?
3. Were women’s experienced fear and pain background factors related to the use of pain assessment and non-pharmacological pain relief methods?

## METHODS

### Sample and setting

We recruited women who had given birth at one of the university hospitals in Finland. The sample size for the present study was calculated based on the number (250) of births per month at the selected hospital; thus, 250 women were recruited to be involved in the study. Women were recruited based on convenience sampling and gave their informed consent to participate in the research while responding to the questionnaire^24^. The inclusion criteria were: 1) birth by vaginal delivery; 2) ability to independently respond to the questionnaire (excluding mentally disabled); and 3) ability to speak and understand the Finnish language.

There were 3309 births, of which 83% were by vaginal delivery, at the selected hospital in 2019. Women usually arrive at the childbirth center from home, but they can also arrive from a local maternity clinic or inpatient ward. The childbirth center is open 24 hours. Pregnant women can come to this center at a pre-scheduled time or if they present various emergency symptoms. Most of the women arrive once labor has started, and midwives take care of them along with doctors. After delivery, a woman and her newborn can transfer to the maternity ward, where mothers can room-in with their newborns.

### Data collection

The data were collected using a validated questionnaire from November 2018 to February 2019. The data collection was organized by two midwives. Participants were asked to respond to the questionnaire after they had given birth in the maternity ward. They had time to answer the questionnaire until they were discharged from the hospital. Data collection continued until 250 questionnaires had been distributed to the women; thus, the recruitment period lasted three months. Of the distributed questionnaires, 207 were returned, with three of these excluded because of missing data. The final sample included 204 women, representing a response rate of 82%.

The participating women were given a printed questionnaire, but also had the possibility to electronically answer (i.e. via mobile phone or tablet) the questionnaire by using a URL or QR code. All of the participants were given adequate information about the research by the two midwives who had recruited the participants. Completed printed questionnaires were returned in sealed envelopes at the end of the data collection period.

### Questionnaire

The P-PAPM (Patients’ perceptions of Pain Assessment and Pain Management in hospitals) questionnaire was based on evidence from earlier studies^1,25-28^ and the opinions of an expert panel including researchers (n=2) and healthcare providers (n=15) specialized in pain management. The questionnaire was pretested on several patients, including parturient women, who also responded to an evaluation form in which they were asked to assess the transparency and clarity of the questionnaire. In addition, they were asked for input concerning the content of the questionnaire and response options. Some minor changes were made based on the results of the pretesting.

The questionnaire included three sections. Section one covered participant demographics, including age, education level, and experiences of fear and pain. In one particular question, women were asked to evaluate their intensity of fear and pain using a Numerical Rating Scale (NRS) from 0 to 10, with 0 representing no fear/no pain and 10 representing severe fear/severe pain^24^. Section two included questions about the implementation of pain assessment (8 items) by the midwives. In addition, it included a question regarding how satisfied the women were with the implementation of pain assessment using a numeral scale from 0 to 10 (0: not at all satisfied, 10: extremely satisfied). Section three included questions about pain management and contained 20 different sub-questions about the use of non-pharmacological pain relief methods during labor. The women replied using a dichotomous-type scale, with the answer choices ‘yes’ or ‘no/I cannot say’. In addition, the women were asked to assess their satisfaction with the use of non-pharmacological and pharmacological pain relief methods using a numeral scale from 0 to 10 (0: not at all satisfied, 10: extremely satisfied).

### Data analysis

Data were analyzed using SPSS Statistics for Windows (version 25.0, IBM, Armonk, NY). Descriptive statistics were used to summarize the participants’ background information along with the responses related to pain assessments and the use of non-pharmacological pain relief methods. The differences between background variables (experienced fear and pain) and variables related to pain assessment and pain management were tested using a chi-squared test. Only statistically significant results (p<0.05) are presented. The intensity of fear was classified as: 0−3 mild, 4−6 moderate, and 7−10 severe. Similarly, the intensity of pain was classified as: 0−3 mild, 4−6 moderate, and 7−10 severe. In addition, satisfaction with the implementation of pain assessment was classified based on the distribution of the data: 0−5 not at all satisfied/minimally satisfied, 6−7 satisfied, 8−9 very satisfied, and 10 extremely satisfied. Satisfaction with the non-pharmacological and pharmacological pain relief methods used during labor was classified in the same way.

### Ethical considerations

A request for ethical approval was sent to the ethics committee of the selected hospital (Ref. No. EETTMK:76/2018). The study complied with the Medical Research Act general provisions^29^ and received permission through the hospital’s own research permit. The Helsinki Declaration^30^ was followed throughout the study. All of the respondents were informed that their participation was voluntary. The pain management nurses who had organized the data collection also verbally explained the purpose of the study to the participants. All of the women replied anonymously to the questionnaire, and it was impossible to associate any personal data to the respondents. The researchers did not meet the respondents. The data were transferred to a private computer and analyzed anonymously^27^.

## RESULTS

### Demographics

**Figure 1:**
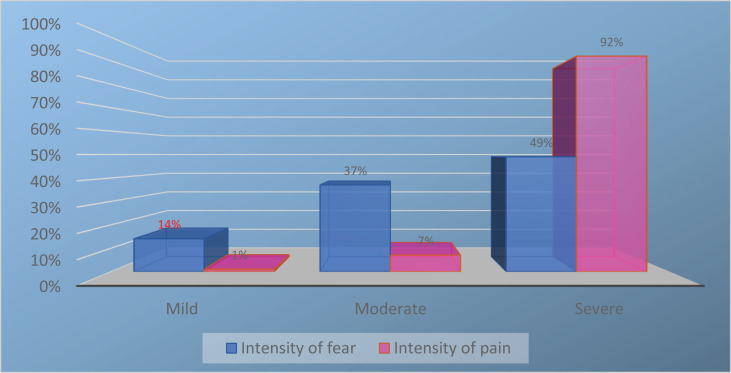
Intensity of fear and pain, as evaluated by the participating women (N=204). Intensity of fear and pain was measured by a numerical rating scale (NRS): no/mild (0–3), moderate (3.5–6.5), severe (7–10)

The participating women had an average age of 31 years (SD: 5.5, range: 20−47). A significant number (n=82; 40%) had completed vocational education/training courses, while one-third (n=61; 30%) had completed college/polytechnic education, one-fifth (n=39; 19%) had completed university education, and about one-tenth (n=19; 9%) had no vocational education. The participants average reported intensity of fear was 6.8 (SD: 2.25), ranging from 1 to 10 on the employed NRS, while the average reported intensity of pain was 8.6 (SD: 1.5), ranging from 3 to 10 (Table 1, Figure 1).

**Table 1:** Women’s experienced fear and pain as background factors related to the implementation of pain assessment

*Pain assessment*		*Fear*	*No fear*	*p*
*I was asked*		*n (%)*	*n (%)*	
About the location of my pain (e.g. what is sore)	Yes	122 (90)	53 (77)	0.011
	No/I cannot say	13 (10)	16 (23)	
To assess my pain with some pain assessment scale	Yes	72 (53)	22 (32)	0.005
	No/I cannot say	63 (47)	47 (68)	
To assess my pain with some pain assessment scale before pain relief	Yes	62 (86)	14 (58)	0.007
	No/I cannot say	10 (14)	10 (42)	
	***Fear scale n (%)***		***p***	
To assess my pain with some pain assessment scale before pain relief	**Mild (0–3)**			
	Yes	7 (100)		
	No/I cannot say	0		
	**Moderate (4–6)**			
	Yes	16 (64)		
	No/I cannot say	9 (36)	0.001	
	**Severe (7–10)**			
	Yes	38 (97)		
	No/I cannot say	1 (3)		
To assess the intensity of the pain with some pain assessment scale	**Mild (0–3)**			
	Yes	2 (100)		
	No/I cannot say	0		
	**Moderate (4–6)**			
	Yes	9 (60)		
	No/I cannot say	6 (40)	0.040	
	**Severe (7–10)**			
	Yes	161 (86)		
	No/I cannot say	26 (14)		

### Implementation of pain assessment

**Figure 2:**
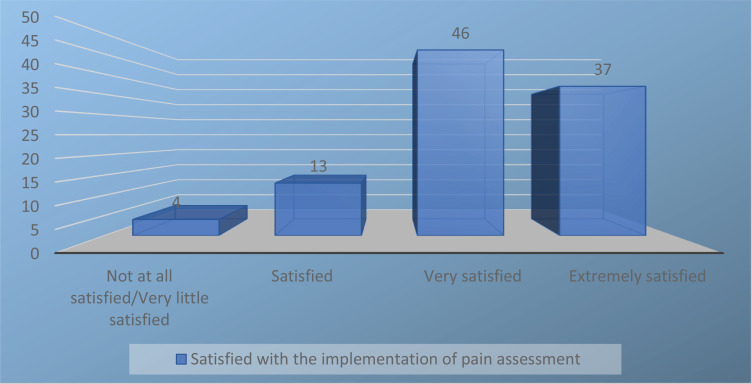
Women’s (N=204) satisfaction with the implementation of pain assessment, as measured by a numerical rating scale (NRS): Not at all satisfied/very little satisfied (0–5), satisfied (6–7), very satisfied (8–9), and extremely satisfied (10)

Less than half of the women were asked to assess the intensity of their pain with some pain assessment scale, and only a few of the women (7%) had an opportunity to influence the choice of which pain assessment scale was used. Most of the women reported that they had been asked about the location (86%), severity (84%), and duration (82%) of their pain, while approximately two-thirds reported being asked about the type of pain they were experiencing. A fifth of the participants reported being taught how to use the pain assessment by a midwife, while one-third of the women reported being assessed using a pain assessment scale before or after receiving pain relief (Table 2). Overall, the women were satisfied with the implementation of pain assessment (mean: 8.7, SD: 1.4, range 5−10) (Figure 2).

**Table 2:** Women’s evaluations of the implementation of pain assessment (N=204)

*Implementation of pain assessment*	*Yes n (%)*	*No/I am not sure n (%)*
I was asked about the location of my pain (e.g. what is sore)	175 (86)	29 (14)
I was asked about the duration of my pain (e.g. when did my pain begin)	168 (82)	36 (18)
I was asked about what type of pain I was experiencing (e.g. drowsy, whistling pain).	142 (70)	62 (30)
I was asked about the severity of my pain (e.g. is the pain mild/moderate/severe)	172 (84)	32 (16)
I was asked to assess the intensity of the pain with some pain assessment scale (e.g. visual analogue scale)	94 (46)	110 (54)
I had an opportunity to influence which pain assessment scale was used	15 (7)	189 (93)
I was instructed/taught how to use the pain assessment scale in my pain assessment	46 (23)	158 (77)
I was asked to assess my pain with some pain assessment scale:		
a) before pain relief (e.g. before the administration of an analgesic)	76 (37)	128 (63)
b) after pain relief (e.g. after the administration of an analgesic)	66 (32)	138 (68)

### Use of non-pharmacological pain relief methods

**Figure 3:**
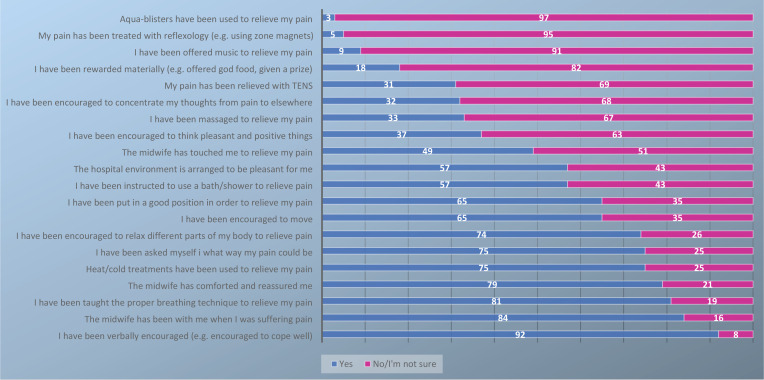
Use of non-pharmacological pain relief methods during labor, as evaluated by the participating women (N=204)

**Figure 4:**
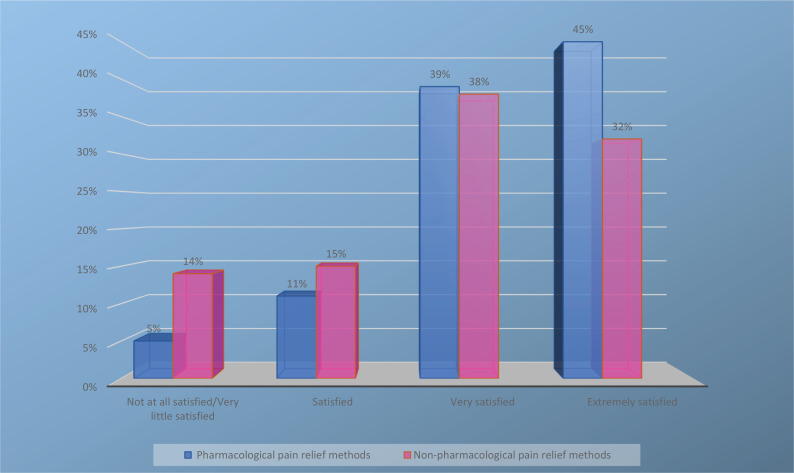
Women’s satisfaction with the use of pain relief methods (pharmacological and non-pharmacological), as measured by a numerical rating scale (NRS): Not at all satisfied/very little satisfied (0–5), satisfied (6–7), very satisfied (8–9), and extremely satisfied (10)

Most of the women reported that their pain had been relieved by encouragement (92%), the presence of a midwife (84%), breathing techniques (81%), and comforting by a midwife (79%). Furthermore, a few of the women reported that they had relieved their pain using aqua blisters (3%), reflexology (5%), or music (9%). A third of the women described how they had been encouraged to concentrate their thoughts away from the pain (e.g. by reading, playing a game, or talking with someone) during labor (Figure 3). Overall, the participants were satisfied with the use of pharmacological pain relief methods (mean: 8.72, SD: 1.7, range 1−10), but not so satisfied with the use of non-pharmacological pain relief methods as measured by an NRS (mean: 8.09, SD: 2.0, range 1−10) (Figure 4).

### Women’s experienced fear and pain related to pain assessment and non-pharmacological pain relief methods

Most of the women (n=135; 66%) experienced fear during labor. Fear was found to be associated with healthcare personnel asking women about the location of their pain (p=0.011), i.e. women who were scared reported being asked about the location of their pain more often than women who were not scared (90% vs 77%, respectively). Experiences of fear were also associated with healthcare personnel assessing the intensity of pain with a pain assessment scale (53% vs 32%, p=0.005).

In addition, fear was associated with the healthcare personnel’s decision to ask a woman to evaluate their pain using a pain assessment scale before pain relief was provided (p=0.007). More specifically, women who were scared were asked to evaluate their pain with a specific pain assessment scale prior to being provided pain relief more often than women who were not scared (86% vs 58%, respectively). It also appeared that women presenting moderate fear were asked to evaluate pain using an assessment scale prior to the provision of pain relief less often than other patients: 4−6 moderate (64%), 0−3 mild (100%), 7−10 severe (97%), p=0.001) (Table 1).

The intensity of pain was significantly associated with the implementation of pain assessment (p=0.040); women whose pain was moderate were not asked that often about the intensity of their pain compared with others: 4−6 moderate (60%), 0−3 mild (100%), 7−10 severe (86%).

The participants’ intensity of fear and pain did not demonstrate any statistically significant associations with the use of non-pharmacological pain relief methods during labor.

## DISCUSSION

Our study produced new knowledge about the implementation of pain assessment and use of non-pharmacological pain relief methods during labor based on the women’s perspectives. The results of this study indicated that most women were asked about their pain verbally without the use of a specific pain assessment scale; more specifically, less than half of the women were asked to assess the intensity of their pain using some pain assessment scale. The women largely did not have the opportunity to influence the choice of pain assessment scale, nor were they often taught how to use the scale for assessing their pain. Additionally, they were seldom asked to evaluate their pain using a pain assessment scale before or after the provision of pain relief. The results of our study confirm what has been reported^8^, i.e. pain assessment is seldom performed in a structured manner and a VAS is not always used to evaluate women’s pain during labor. In our study, the decision not to use a pain assessment scale might be explained by the continuous monitoring of a mother’s contractions via cardiotocography during labor. In addition, the implementation of a pain assessment scale requires continual interaction between the midwives and the women^31^, and as such would require additional time for the midwife to teach the patient how to use a selected pain assessment scale. However, it would be important for the women to be involved in the delivery process by having the opportunity to influence which pain assessment scale is used. In our study, women who experienced fear were offered more frequently a pain assessment tool for evaluating their pain than women who did not experience fear. In our study, about half of the women experienced severe fear, while only 14% of the women in a previous study^32^ (853988 pregnant women) experienced severe fear, in a global context. However, in our study, experienced fear and pain were not associated with the use of non-pharmacological pain relief methods during labor. Pain is one of the most important concerns during labor when it is essential to involve women in their pain assessment and use pain assessment scales based on their individual choices. In addition, pain assessment forms a basis for effective pain relief^6^.

In our study, most of the women received some non-pharmacological pain relief during labor, whereas in a Swedish study by Robertson and Johansson^23^ between 17% and 35% of the women had received some non-pharmacological pain relief. In our study, the prevalence of non-pharmacological pain relief use was higher than what has been reported for Finland (42%)^22^. However, the methods that required more than the midwife’s contribution (e.g. music, aqua blisters, reflexology) were seldom offered for pain management to the women in labor. This is consistent with the results of a previous study^14^ that found relaxation, movement, and a midwife’s psychological support, to be the non-pharmacological pain relief methods used most often during labor, with half of the women using these methods. In our study, most of the women were supported by midwives and that might have affected the women’s satisfaction with non-pharmacological and pharmacological pain relief methods. A previous study, of 22000 participants from 12 countries, found support from midwives, doula or chosen company to be the most effective strategies for a positive experience of childbirth^19^.

It is surprising that music was not offered as an obvious form of pain relief to the participants of our study. Most women had their own headphones in the maternity ward, and music could easily offer some distraction during painful situations in labor^26^. In addition, music has been found in previous studies^33,34^ to be an easy and useful method to relieve pain and anxiety during labor. The lack of versatile non-pharmacological pain relief methods might be explained by the widespread use of analgesics, such as epidurals, which are shown to be the most effective at handling labor pain^1^. According to previous statistics^22^, most Finnish women (92%) use some pain relief during labor, with the most common pharmacological remedies being nitrous oxide (54%), epidurals (50%), and spinal block (20%). Finland is one of several countries characterized by low neonatal and maternal mortality^35^ as a result of the high-quality, evidence-based care in maternity hospitals^36^. As such, it is possible that certain hospital regulations and policies might explain why non-pharmacological pain relief methods are not used as often as pharmacological interventions^14^. However, based on both the evidence presented in this article and previously published results, non-pharmacological pain relief methods may offer better satisfaction and a sense of pain control during labor than standard care^1,14^.

In our study, women were first asked the prevalence of used non-pharmacological pain methods during their labor, after which they were queried about their satisfaction with these non-pharmacological methods. They were not asked, however, to describe or speculate the reason why women were not offered non-pharmacological pain relief methods by midwives. According to Almushait et al.^14^, most women were satisfied with their labor experiences, while only half of them desired non-pharmacological relief. Previous research has shown that women are not always well prepared for labor and may not be aware of the pain relief methods available to them^12^.

Our study indicated that assessing women’s pain is challenging in the clinical context, as reported in another study^9^. It is interesting that women who were scared were asked to assess their pain more often than women who did not show fear during labor. Experiences of fear have been shown to be strongly associated with the preference for caesarean section^37^ as well as the duration of labor^38^; hence, the midwives may have prioritized the care of women who seemed to be more stressed, and could have offered a pain assessment to them. Even though the participating women reported that pain assessment scales were not often used by the midwives, the women were nevertheless satisfied with the pain assessment they experienced. Another study^9^ reported that women want to focus on their active labor and for this reason felt unable to describe or rate their pain. Our study also showed that the presence of midwives was no guarantee that a pain assessment would be conducted or that a specific pain assessment scale would be used during assessment.

In our study, women who had moderate pain were not often asked about their pain. During labor, pain varies from woman to woman, with some individuals able to handle the pain without a need for any intervention^1^. This might explain why midwives were less active in asking women demonstrating moderate pain about their current situation. It was surprising that neither the intensity of fear nor pain was statistically significantly associated with the use of non-pharmacological pain relief methods during labor. According to Lundgren and Dahlberg^39^, women’s perceptions of pain are affected by physiological and/or psychological issues (e.g. fear or anxiety). In the selected hospital, women could easily receive pharmacological pain relief, and this might have affected the use of non-pharmacological pain relief methods.

It is important to develop midwife policies and practical work, and this study provides novel insight into women’s perceptions of pain assessments and non-pharmacological relief during labor. The results suggest that maternity centers must ensure that all midwives are knowledgeable about diverse non-pharmacological pain relief methods, as offering these methods can improve women’s experiences of childbirth. This is consistent with the research by Lowe^3^, which highlights that labor and birth include intense physical, emotional, and spiritual elements that may be critical to an individual woman’s experience of this important life event.

### Strengths and limitations

We chose to describe the perception of women who had recently given birth as we felt that they would be the best informants regarding the implementation of pain assessment and use of non-pharmacological pain relief methods during labor. The good response rate (82%) attests to how motivated these women were to describe their experiences. However, the study also included some limitations. The sample size for the present study was calculated based on the number (250) of births per month at the selected hospital, but it took about three months to collect the data. This may have influenced the representatives of the sample and the generalization of our results. The participants were asked to respond to the questionnaire soon after labor. In addition, people often tend to respond according to social expectations when responding to a questionnaire. Therefore, the reliability of the study may have benefitted from the collection of diverse data, i.e. conducting individual interviews or observing women during labor. It is important to state that the presented results should be generalized with caution because the results only represent one university hospital of Finland. In any case, the results are in line with what has been reported in other studies^8,12,14^. This study was part of a large pain assessment and management project at the selected hospital; thus, specific background questions that would be relevant to women in labor (nulliparous/multiparous, which pharmacological pain relief methods were used during labor) are missing. The data collected through these types of questions could have provided more insight into the associations between both non-pharmacological and pharmacological methods for pain relief and women’s perceptions of how pain assessment and management were implemented during labor^6,10,12^.

## CONCLUSIONS

According to our findings, women’s pain was rarely evaluated by a pain assessment scale but was rather based on the midwives asking women about the intensity of their pain. The results also reveal that non-pharmacological pain relief methods, e.g. aqua blisters, reflexology, and music, which require more than the presence of a midwife, were inadequately provided based on the women’s perceptions during labor. The presence of a midwife was the most used non-pharmacological pain relief method. Women’s experienced fear and pain were related to the implementation of pain assessment but not to the use of non-pharmacological pain relief methods which requires more research in the future. In addition, further research is needed to determine how effective these methods are at providing pain relief in comparison to commonly used approaches, such as analgesics.

## Data Availability

The data supporting this research cannot be made available for privacy reasons.
